# Differentiation between Hydrolytic and Thermo-Oxidative Degradation of Poly(lactic acid) and Poly(lactic acid)/Starch Composites in Warm and Humid Environments

**DOI:** 10.3390/ma17153683

**Published:** 2024-07-25

**Authors:** Victoria Goetjes, Jan-Christoph Zarges, Hans-Peter Heim

**Affiliations:** Institute of Material Engineering, Polymer Engineering, University of Kassel, Mönchebergstr. 3, 34125 Kassel, Germany

**Keywords:** biopolymers, PLA, starch, durability, aging, hydrolysis, oxidation

## Abstract

For the application of poly(lactic acid) (PLA) and PLA/starch composites in technical components such as toys, it is essential to know their degradation behavior under relevant application conditions in a hydrothermal environment. For this purpose, composites made from PLA and native potato starch were produced using twin-screw extruders and then processed into test specimens, which were then subjected to various one-week ageing processes with varying temperatures (23, 50, 70, 90 °C) and humidity levels (10, 50, 75, 90%). This was followed by mechanical characterization (tensile test) and identification of degradation using Gel Permeation Chromatography (GPC), Thermogravimetric Analysis (TGA), Fourier Transform Infrared Spectroscopy (FTIR), and Nuclear Magnetic Resonance spectroscopy (NMR). With increasing temperature and humidity, there was a clear degradation of the PLA, which could be reduced or slowed down by adding 50 wt.% starch, due to increased crystallinity. Hydrolysis was identified as the main degradation mechanism for PLA and PLA/starch composites, especially above the glass transition temperature, with thermo-oxidative degradation also playing a subordinate role. Both hydrolytic degradation and thermo-oxidative degradation led to a reduction in mechanical properties such as tensile strength.

## 1. Introduction

Poly(lactic acid) (PLA) is considered the best-known representative of bio-plastics. Currently, PLA is mainly used in the packaging and medical sectors [1]. For use in more demanding applications, in which high resistance to elevated humidity and temperatures is required in particular, the behavior under hydrothermal conditions must be investigated first.

PLA is considered a material susceptible to hydrolysis when exposed to moisture or water [2,3,4,5,6,7,8,9,10,11,12,13,14,15,16]. Water diffusing into the polymer causes random chain scission, leading to a reduction in molecular weight [5]. With increased chain mobility due to elevated temperatures, the interaction of temperature and humidity increases and hydrolysis is accelerated [17]. In addition, elevated temperatures and an oxygen-containing atmosphere ensure the occurrence of thermo-oxidative degradation processes [18,19]. Previous studies already showed that after 504 h at 70 °C and 50% relative humidity (RH), significant degradation of PLA took place, leading to complete damage of the material. Improving the resistance of PLA is therefore imperative if it is to be used in technically advanced applications like toys.

One possibility for optimization is filling PLA with native starch, because the addition of starch can increase the crystallinity of PLA and thus increase its resistance, especially to hydrolytic degradation [20,21,22,23]. The starch can be obtained from various raw materials such as maize, rice, or potatoes. In addition, the use of starch as a filler can significantly reduce the material’s carbon footprint [24]. Nevertheless, the use of the filler brings disadvantages: First, the mechanical properties of the material decrease with increasing starch content and it becomes more brittle, showing reduced tensile strength and elongation at break [25,26]. Second, the starch increases the water absorption of the composite [23,27]. This increased water absorption in turn promotes the hydrolytic degradation of PLA and can additionally lead to physical aging processes in the form of swelling and shrinkage effects [23,28,29,30,31,32,33]. The extent to which the addition of starch to PLA therefore has a positive or negative effect on applications in hydrothermal environments has not been definitively clarified.

In the course of the investigation carried out, samples of PLA and PLA filled with 50 wt.% potato starch were subjected to one-week storage at different temperatures and levels of humidity and subsequently characterized. The aim of the investigations was to identify the difference between physical and chemical aging processes, as well as to distinguish different chemical degradation mechanisms. Only through a sufficient understanding of the degradation mechanisms can ageing be prevented and the use of PLA and PLA/starch composites in durable products be realized. Therefore, in the following, the degradation mechanisms of PLA are presented first, followed by the mechanical and structural characterization of the aged composites.

## 2. Aging Mechanisms of PLA

**Figure 1:**
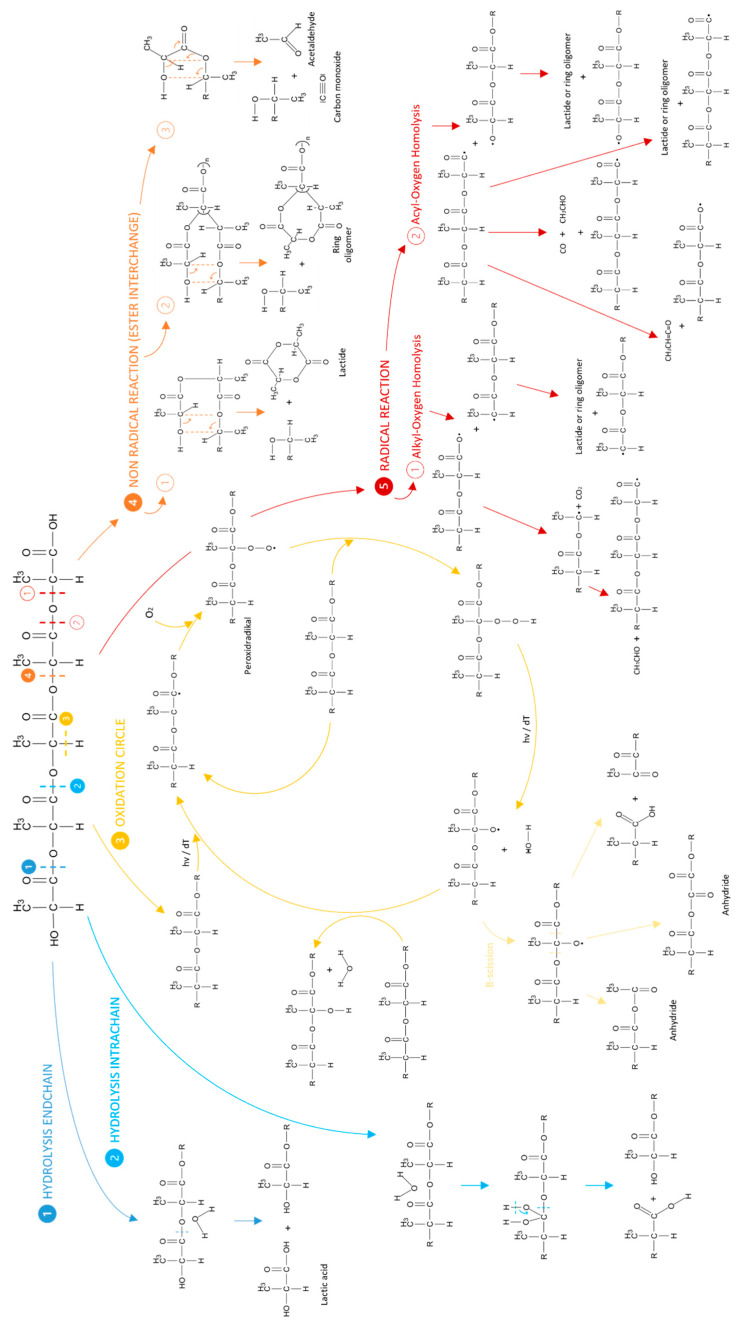
Overview of the degradation mechanisms of PLA under hydrothermal conditions [2,5,7,9,18,34,35,36,37,38,39,40,41,42].

The aging of PLA and PLA composites is a highly complex interaction of different mechanisms. In addition to reversible physical effects, irreversible chemical aging processes also occur. In warm and humid environments, these processes extend to hydrolytic and thermo-oxidative degradation. To distinguish between the different types of degradation, it is essential to know the mechanisms involved. Figure 1 shows an overview of the most important degradation mechanisms of PLA under hydrothermal conditions.

Due to the hygroscopic nature of PLA, hydrolysis of the ester group is the main degradation mechanism of PLA [34]. The hydrolysis can occur either at the end of the chain (1) or within the chain (2), without a difference in the mechanism. The hydrolytic degradation of PLA can be accelerated by elevated temperatures and acidic or basic environments. The formation of lactic acid during hydrolytic degradation accelerates itself by creating an increasingly acidic environment [2,5,7,9,35,36,37,38].

In non-inert environments, thermo-oxidative degradation can occur in addition to hydrolysis, which in turn can be subdivided into three mechanisms. For oxidative degradation (3), a radical must first be formed as a result of elevated temperature or the impact of photon energy with the splitting off of a hydrogen atom. In the presence of oxygen, a peroxide radical is formed, which further follows the autoxidation cycle. The peroxide thus takes up hydrogen from another macromolecule, resulting in the formation of a new radical [34,39,40]. This in turn can be further degraded by β cleavage, with two of the three mechanisms producing anhydrides [41].

In addition, three different nonradical reactions (4) were observed as a result of thermo-oxidative degradation. The so-called ester interchanges were identified by McNeill and Leiper [42] and observed in inert atmospheres, but can also occur in the presence of oxygen. Compared to the radical reactions (5), the non-radical reactions have a relatively low activation energy [18,42]. Depending on the location of the rearrangements, the degradation products can differ between a lactide molecule, the ring oligomer, and acetalaldehyde and carbon monoxide.

The end products are formed the same way in the radical reactions (5), which can be divided into alkyl oxygen homolysis and acyl oxygen homolysis. In addition, five different macroradical types are formed. Acyl oxygen homolysis has a lower activation energy than alkyl oxygen homolysis. The radical reactions were observed by McNeill and Leiper [42] at temperatures above 270 °C only, but for the purpose of completeness they are nevertheless listed here [18,42].

## 3. Materials and Methods

### 3.1. PLA and Potato Starch

PLA Luminy^®^ L130 from TotalEnergies Corbion (Gorinchem, The Netherlands), which has a melting temperature of 175 °C and a density of 1.24 g/cm^3^, was used for the tests [43].

In addition, a native potato starch of the type Superior from Emsland Stärke (Emlichheim, Germany) was used, as potato starch has an oval shape and is therefore very well suited as a bio-based filler and is locally available. A starch content of 50% was used, as the highest possible filler content was to be realized without causing significant restrictions in processability due to the reduction in the carbon footprint. The starch used consists of amylose (22%), amylopectin, water, ash, phosphorus, fat, and protein [44].

### 3.2. Preparation of the Composites and Samples

Samples of pure PLA as reference and samples with 50 wt.% starch were produced. For this purpose, composites were first compounded by twin-screw extrusion (ZSE 18 HPe, Leistritz Extrusionstechnik GmbH, Nuremberg, Germany) and subsequently produced by an injection molding process to form type 1A specimens according to DIN EN ISO 527-2 [45]. Prior to compounding, both PLA (100 °C, 6 h) and potato starch (105 °C, 8 h) were pre-dried. PLA was melted via the main feeder up to zone three by conveying and kneading elements. Starch was added via a side feeder in zone 4, and mixing elements in zones 6 and 7 were used to mix the two components and degas them. A throughput of 3 kg/h was realized at a speed of 200 rpm. The temperature was increased from 170 to 200 °C constantly across the 8 zones. Previous investigations showed that the choice of process parameters allows sufficient compounding without degrading the starch [23,25]. The test specimens were produced on an Allrounder 320C injection molding machine (Arburg GmbH + Co. KG, Lossburg, Germany). Again, the composites were pre-dried at 100 °C for 6 h. Injection molding was performed at a maximum injection pressure of 1040 bar (filled composite) and a holding pressure decreasing from 700 to 500 bar for 25 s. The total cycle time was approximately 98 s. The temperature was increased from 200 to 215 °C in 5 °C steps in zones 1 to 4 and then kept constant until the nozzle. The mold temperature was 30 °C [23].

### 3.3. Ageing in Warm and Humid Environments

**Figure 2:**
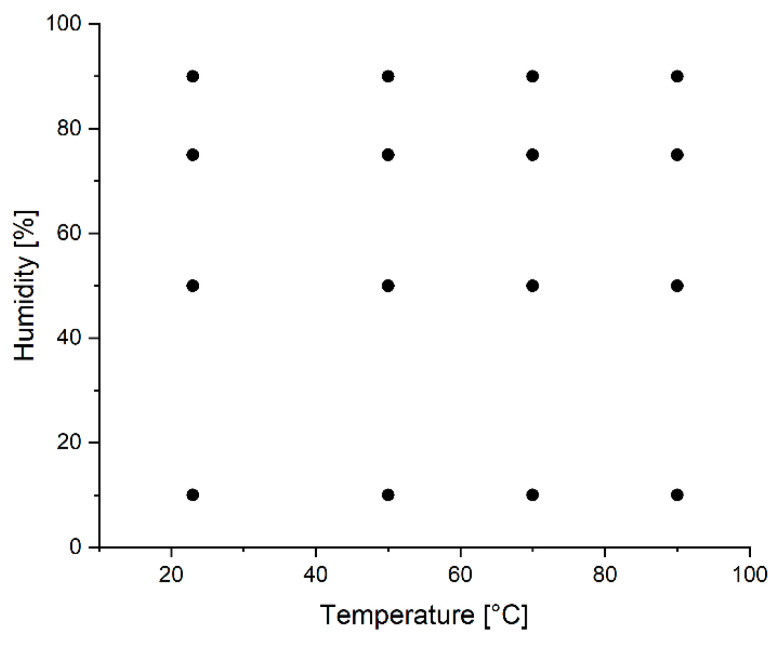
Illustration of the experimental design—combination levels of temperature and humidity.

To identify the predominant degradation mechanisms of PLA and PLA–starch composites under the influence of elevated temperatures and humidity levels, the test specimens were exposed to different environmental conditions (Figure 2) for 48, 96, and 168 h each in a climatic test chamber. Temperatures of 23, 50, 70, and 90 °C and relative humidity levels of 10, 50, 75, and 90% RH were used. Prior to further testing and characterization, all aged specimens were subjected to conditioning in a standard climate according to DIN EN ISO 291 [46] at 23 °C and 50% RH for at least 168 h.

### 3.4. Tensile Test

For the mechanical characterization, the aged and conditioned specimens were subjected to a tensile test according to DIN EN ISO 527-2 [45], and Young’s modulus, tensile strength, and maximum elongation were determined. The tests were performed on a UPM 1446 testing machine from Zwick Roell (Ulm, Germany) with 5 specimens of each batch at a test speed of 5 mm/min. Significance analysis was performed using ANOVA with the Tukey test at a significance level of 0.05 using Origin software (2021b). The contour plots used were created with Origin software and smoothed with an XYZ interpolation using the Thin Plate Spline (TPS). The “Total points increase factor” was 100 and the smoothing parameter was 0.05.

### 3.5. Gel Permeation Chromatography (GPC)

In order to determine whether chemical aging, i.e., polymer degradation, has occurred, GPC analyses of the unfilled and filled PLA were performed by the Fraunhofer-Institute for Applied Polymer Research IAP (Potsdam-Golm, Germany). The measurement was performed according to DIN 55672-1 [47] using the eluent trichloromethane (TCM) at 25 °C. The number average molecular weight (Mn), weight average molecular weight (MW), and molecular weight distributions were determined. Significance analysis was performed using ANOVA with the Tukey test at a significance level of 0.05 using Origin software. The contour plots used were created with identical parameters as for the tensile test.

### 3.6. Thermogravimetric Analysis (TGA)

To rule out pure thermal degradation, TGA of the unaged pure PLA sample and the unaged PLA–starch sample was carried out on the TGA Q500 (TA Instruments, New Castle, DE, USA). The measurements were carried out under a synthetic air atmosphere. The 30 mg samples were heated from 25 to 800 °C at a heating rate of 10 Kmin^−1^.

### 3.7. Fourier Transformation IR Spectroscopy (FTIR)

To describe the degradation of the PLA and the PLA–starch composites, FTIR measurements were carried out using the IRAffinity-1S from Shimadzu (Duisburg, Germany) with a zinc–selenium crystal. The measurement resolution was 2 cm^−1^ in the wavelength range between 600 cm^−1^ and 4000 cm^−1^.

### 3.8. NMR Spectroscopy

The degradation of PLA was confirmed by ^1^H-NMR and ^13^C-NMR spectroscopy. For each sample, 6 mg of the solid product was dissolved in 0.5 mL of deuterated chloroform (CDCl3). The ^1^H-NMR and ^13^C-NMR spectra were recorded with a Varian (Palo Alto, CA, USA) NMRS-500 and MR-400 MHz NMR spectrometer operating at 500 and 400 MHz. The spectra were processed using MestReNova software (version 14.2.0-26256).

## 4. Results

### 4.1. Influence of Warm and Humid Environment on Mechanical Properties of PLA and PLA–Starch Composites

**Figure 3:**
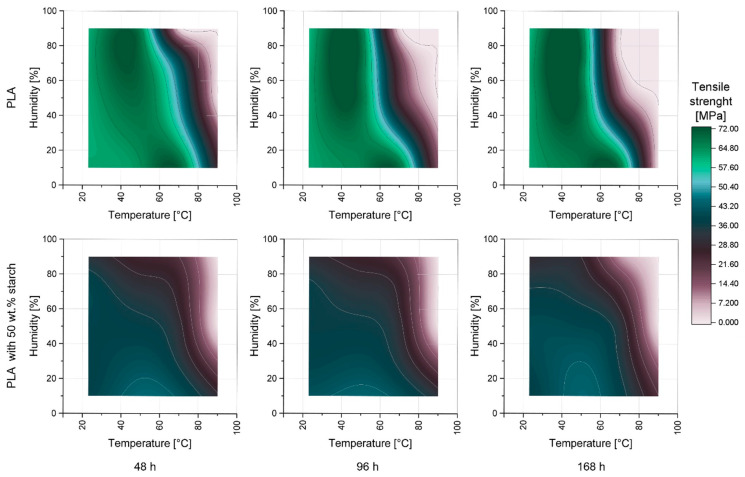
Tensile strength of PLA and PLA–starch composites as a function of temperature, RH, and storage time.

Figure 3 shows the tensile strength of PLA (top) and the composite of PLA and 50 wt.% starch (bottom) as a function of relative humidity (RH), temperature, and storage time. The mechanical properties of the specimens after 168 h storage time are also shown in Table 1 and Table 2. With the addition of the starch, the reference samples (23 °C and 10 % RH) already show significant embrittlement of the test specimens regardless of ageing. This observation has already been demonstrated in numerous studies [23,25,26].

For the samples made of pure PLA, after 168 h at room temperature (23 °C) with increasing RH, initially there was no significant change in tensile strength. Increasing the temperature to 50 °C showed a significant increase in tensile strength up to 71.08 MPa at 75% RH, independent of RH. At 10% RH, increasing the temperature to 70 °C resulted in a further increase in tensile strength from 67.71 MPa at 50 °C to 70.70 MPa. On the other hand, a further increase in temperature at 10% RH to 90 °C led to such severe damage to the test specimens that no further testing could be performed. For the test specimens at 50% RH, a temperature increase from 50 to 70 °C resulted in no increase in tensile strength compared to 10% RH, but a significant decrease to 14.33 MPa. This relationship could also be observed at 75 and 90% RH when the temperature was increased to 70 °C, although the damage here was so severe that testing was no longer possible. At 90 °C, no mechanical testing could be performed due to the excessive damage independent of RH.

Examining the influence of storage time on tensile strength, after 48 h, initially only high RH (>80%) at temperatures above 70 °C or temperatures above 80 °C at RH above 30% were shown to be particularly critical. With increasing storage time, the critical range widened significantly.

For the composites with 50 wt.% starch, some differences in the behavior of the pure PLA could be seen. Already at low temperatures and RH, a significantly lower tensile strength could be observed compared to identically stored pure PLA samples. In addition, at both 23 and 50 °C, a significant influence of increasing humidity could be seen, leading to a reduction in tensile strength. Furthermore, PLA degradation seemed to be slowed down or inhibited by the addition of starch, as the critical area (light pink), where such large damage occurs that no testing could take place, was reduced.

Initially, increases in tensile strength were observed for both filled and unfilled samples, although these only occurred in the range of low RH for starch-containing samples. This observation can be attributed to an increase in crystallinity of the PLA as a result of the increased temperature. This leads to increased mobility of the chains and thus rearrangement of these in crystalline structures, which increases their mechanical properties [48].

As demonstrated in previous studies, elevated temperatures (especially above T_g_) and high RH over extended periods lead to the degradation of PLA [23,49,50]. This degradation, which may result from both hydrolytic processes and thermo-oxidative degradation, is responsible for the substantial reduction in tensile strength and the embrittlement of PLA samples in humid and warm environments [8,10,11,12,13,14,15,16,51,52,53]. The addition of starch to PLA can increase the crystallinity of PLA, thus enhancing its resistance to hydrolytic and thermo-oxidative degradation [20,21,23,25,54]. However, starch in humid environments also leads to the occurrence of physical aging and crack formation, which contribute to a decrease in mechanical properties [23]. Therefore, the reduction in the mechanical properties of starch-containing samples is not only attributable to polymer degradation, which is why the polymer degradation was subsequently identified.

### 4.2. Influence of Warm and Humid Environment on the Degradation of PLA and PLA–Starch Composites (GPC)

**Figure 4:**
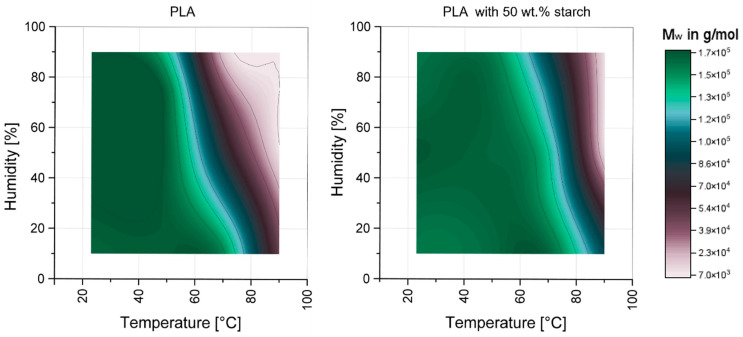
Weight average molecular weights of aged PLA (**left**) and PLA from PLA–starch composite (**right**) after 168 h as a function of temperature and RH.

To identify the influence of the hydrothermal environment on the chemical aging of PLA and PLA–starch composites, GPC measurements were performed. Figure 4 shows the mean molar mass of the aged samples. For pure PLA, at 23 °C and increasing humidity, there is initially a statically significant increase in mean molar mass, followed by a small reduction. At 50 °C, a reduction in the molar mass is seen with increasing humidity, and only between 75 and 90% a significant decrease can be observed. For temperatures above T_g_ (70 °C and 90 °C), a significantly reduced molar mass is shown with increasing humidity. At 90 °C, no further reduction in molar mass can be observed above 50% RH.

For the PLA, which was used as the matrix material in the PLA–starch composite, similar relationships were found, with improved resistance to elevated temperatures (70 °C). This can be attributed to the nucleating effect of the starch particles [20,21,22,23,25]. At particularly critical conditions (90 °C at min. 50% RH), the increased crystallinity of the PLA is no longer sufficient to reduce degradation, and an “equilibrium state” is also established here. The initial increase in molar mass at 23 °C is due to crosslinking of the PLA [55,56].

With increasing temperature, as well as with increasing humidity, aging and thus irreversible degradation of the PLA takes place. Especially elevated temperatures of at least 70 °C at elevated humidity levels of at least 50% are critical. In the following, various investigations were carried out in order to identify the cause of the degradation processes or the underlying mechanisms.

### 4.3. Thermal Stability of PLA and PLA–Starch Composites (TGA)

First, both unaged PLA and unaged PLA–starch composite were subjected to TGA measurement to rule out thermal degradation during the aging performed.

**Figure 5:**
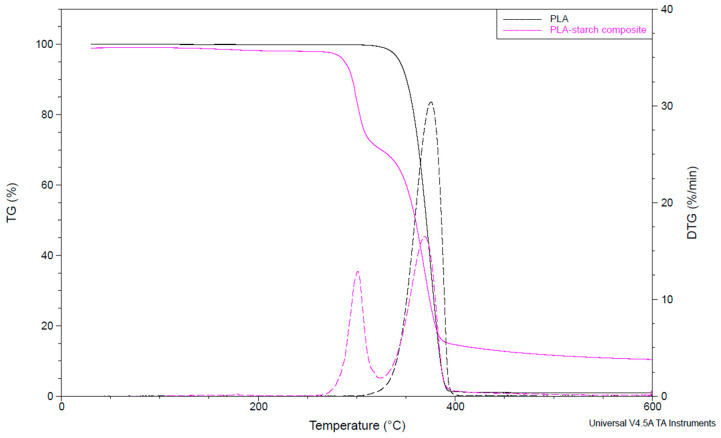
TGA and DTG (dot lines) curves of PLA and PLA–starch composite.

Figure 5 shows the mass reduction in the unaged materials as a function of temperature. As expected, the samples below 100 °C can be described as thermally stable, so that thermal degradation mechanisms can be excluded. The thermal degradation of the pure PLA starts at about 320 °C and follows a one-step process. The composite degradation occurs in two stages from approx. 280 °C, whereby it can be assumed that the starch is degraded first, because of its lower thermal stability [57].

### 4.4. Investigation of Structural Changes in PLA and PLA–Starch Composites by Hydrolysis and Thermo-Oxidative Degradation Using FTIR

**Figure 6:**
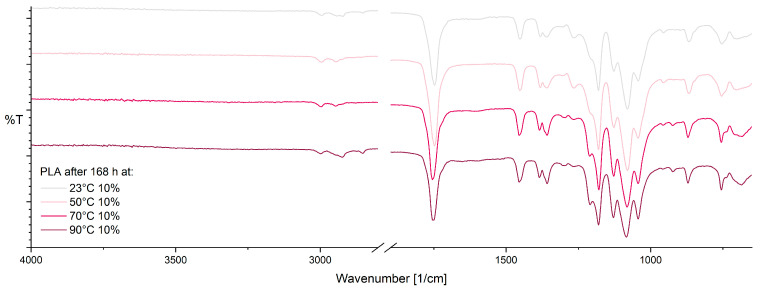
ATR-FTIR spectra of PLA after 168 h at 10% RH as a function of temperature.

FTIR spectroscopy was used to identify changes in the chemical structure as a result of aging. Figure 6 shows the spectra for aged PLA samples after 168 h at varying temperatures and 10% RH. First, a change in the peaks at 1360 cm^−1^, 1265 cm^−1^, and 1130 cm^−1^ can be observed, which is due to a change in the vibrational modes of CH and C-O-C groups as a result of thermo-oxidative degradation [58]. In addition, there is an increase in the peak at 1040 cm^−1^. The increase in the peak at 1210 cm^−1^ suggests the formation of new C=O bonds, which may result from thermo-oxidative degradation [58]. The reduction in the peak at 955 cm^−1^ (amorphous part) and the increase in the peak at 921 cm^−1^ (crystalline part) show an increase in the crystallinity of PLA with increasing temperature [38,59]. Changes in the C=O peak around 700 cm^−1^ can be attributed to an oxidation reaction [60].

**Figure 7:**
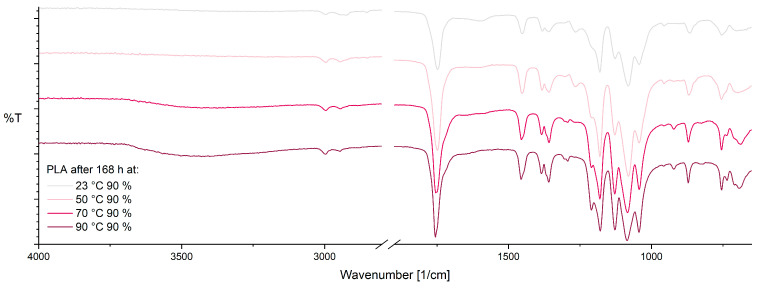
ATR-FTIR spectra of PLA after 168 h at 90% RH as a function of temperature.

The spectra of PLA after 168 h at varying temperature at 90% RH are shown in Figure 7. In the range between 3700 cm^−1^ and 3050 cm^−1^, a clear peak is formed with increasing temperature, attributed to potential hydrolysis products such as lactic acid [61]. In the context of thermo-oxidative degradation, there is a change in the vibrational modes of CH and C-O-C groups, resulting in changes in the peaks at 1362 cm^−1^, 1265 cm^−1^, and 1128 cm^−1^ as observed here. Also, the formation of a new band at 1210 cm^−1^ is observed, which speaks for the formation of new C=O groups, e.g., by thermo-oxidative degradation [58]. Oxidation reactions also cause the changes in the C=O peak at 700 cm^−1^ [60]. The formation of a small shoulder around 1710 cm^−1^ can also be related to thermo-oxidative degradation processes, in particular the formation of Acetaldehydes [62]. As a result of increasing aging, there is an increase in crystallinity, which is why an increase in the crystalline fraction at 921 cm^−1^ and a decrease in the amorphous fraction at 955 cm^−1^ can be observed [38,59,63].

**Figure 8:**
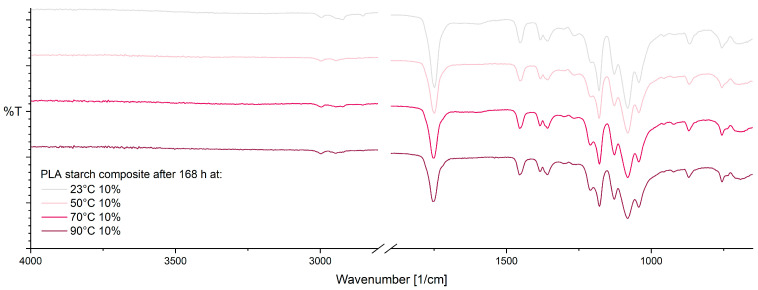
ATR-FTIR spectra of PLA–starch composite after 168 h at 10% RH as a function of temperature.

For PLA–starch composite, after 168 h at 10% RH (Figure 8), only minor changes in the FTIR spectra with increasing storage temperature are evident. As with pure PLA, an increase in crystallinity (921 cm^−1^ and 955 cm^−1^) can be observed with increasing temperature due to annealing effects [38,59]. Supplementary, slight changes in the vibrational modes of CH and C-O-C groups at 1210 cm^−1^ and 1265 cm^−1^ are shown, as well as the formation of a small shoulder at approx. 1710 cm^−1^. The mentioned changes could be caused by thermo-oxidative degradation of PLA [38,58,59,62].

**Figure 9:**
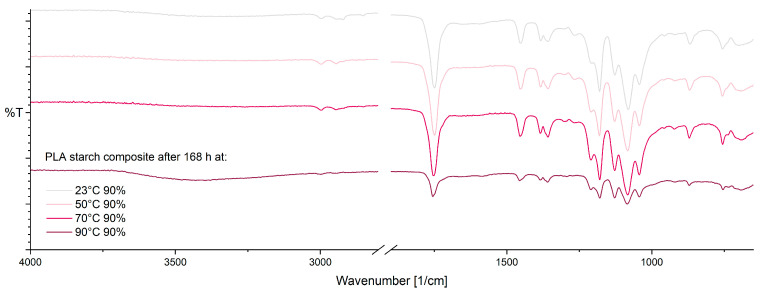
ATR-FTIR spectra of PLA–starch composite after 168 h at 90% RH as a function of temperature.

Even at 90% RH (Figure 9), the PLA–starch composite shows an increase in crystallinity with increasing temperature (921 cm^−1^ and 955 cm^−1^), due to hydrolysis [38,59]. As with 10% RH, changes in the vibrational modes at 1210 cm^−1^ and 1265 cm^−1^, possibly due to thermo-oxidative degradation, are evident [38,58,59]. In the range from 3050 cm^−1^ to 3700 cm^−1^, the formation of a band can be observed, which could be due to hydrolytic degradation [58,61].

The FTIR spectra illustrate the degradation processes and thus chemical structural changes that result from aging in hydrothermal environments, but do not allow clear differentiation of the mechanisms in view of the expected degradation mechanisms (Figure 2).

### 4.5. Differentiation between Hydrolytic and Thermo-Oxidative Degradation of PLA Using NMR Spectroscopy

**Figure 10:**
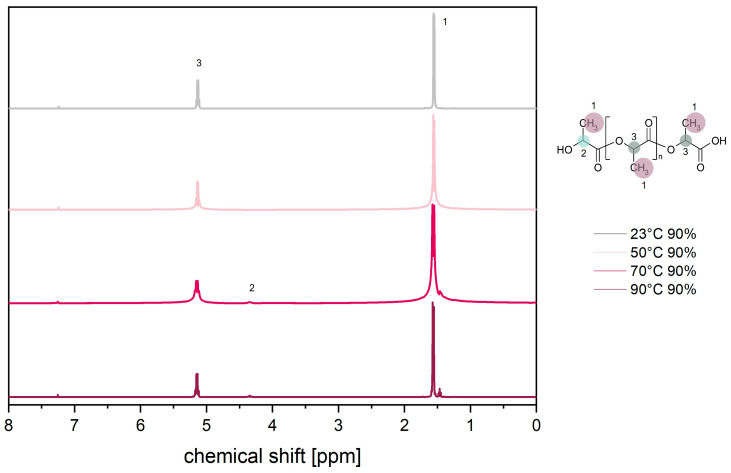
^1^H-NMR spectra of PLA after 168 h at 90% RH as a function of temperature.

To obtain further insights into the degradation mechanisms, both ^1^H and ^13^C NMR spectra of the aged (168 h) PLA samples were recorded. Figure 10 shows the ^1^H-NMR spectroscopy of PLA at different temperatures and 90% RH.

Initially, the expected peaks of CH_3_ protons at 1.6 ppm (1) and CH protons at 5.2 ppm (3) can be seen. With increasing temperature and thus increasing degradation, the number of protons in the chain edge region (2) increases and the number of protons within the repeat unit (3) decreases. Consequently, the formation of a new peak at 4.3 ppm (2) can be observed. These changes are due to chain scission by hydrolytic degradation [64,65,66,67]. In addition, another small peak develops below the CH_3_ peak.

**Figure 11:**
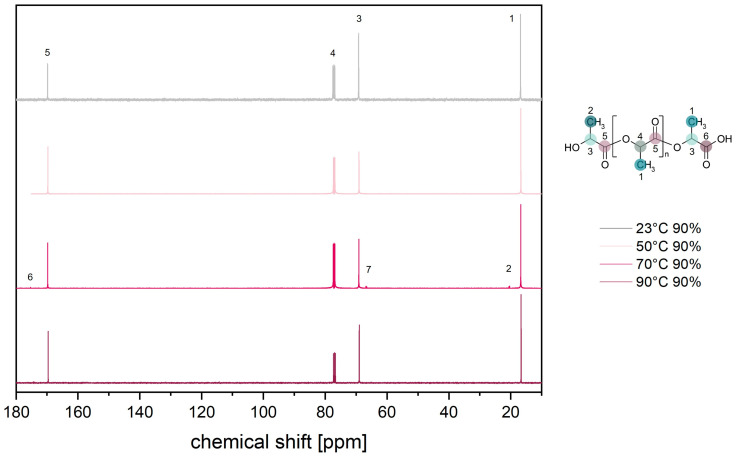
^13^C-NMR spectra of PLA after 168 h at 90% RH as a function of temperature.

Figure 11 shows the ^13^C-NMR spectroscopy of PLA at different temperatures and 90% RH. Initially, the characteristic peaks of the methyl group appear at 17 ppm (1), the methine group at 69 ppm (3) and 77 ppm (4), and the carbonyl group at 170 ppm (5). As already observed in the ^1^H-NMR spectra, the increasing temperature leads to degradation of the PLA and thus to chain scission. Consequently, there are more edge units in relation to repeat units and new peaks of the methyl group at 20 ppm (2). There is also a new peak of the carboxyl group at 174 ppm (6), as well as a change in the ratio of the methine groups (3 and 4). The changes are due to hydrolytic chain degradation (see Figure 2) [67,68,69].

In addition, other small changes in the peaks can be seen, which may be due to thermo-oxidative degradation processes, including the formation of the peak (7) (Figure 11).

## 5. Conclusions

In this paper, the degradation of PLA and PLA–starch composites in a hydrothermal environment was investigated. The aim of the investigations was to identify the existing degradation mechanisms and to characterize the influence of starch on the degradation of PLA.

The conducted investigations illustrate the interactions that take place between the combined influence of temperature and humidity on PLA and PLA–starch composites. Firstly, both increased humidity and temperature lead to a reduction in the mechanical properties up to complete damage of the PLA, which can be attributed entirely to chemical degradation. When starch is added, the chemical degradation can be partially reduced at elevated temperatures, but at the same time, the starch leads to increased water absorption [23], which in turn leads to cracks and a reduction in mechanical properties due to mechanical damage. TGA, FTIR, and NMR measurements were carried out to identify the degradation mechanisms. It was shown that no thermal degradation takes place and that the reduction in molecular weight is due to hydrolytic and thermo-oxidative processes. Both the NMR spectra and the FTIR spectra show clear signs of hydrolysis processes. Nevertheless, there are also signs of thermo-oxidative degradation processes, such as changes in the FTIR spectra around 1710 cm^−1^, which indicate the formation of aldehydes [62]. In general, it is evident that the primary degradation mechanism within the temperature range of 23 to 90 °C and relative humidity between 10 and 90% is attributed to hydrolysis. At elevated temperatures, thermo-oxidative degradation processes are additionally observed, albeit assuming a subordinate role.

## Figures and Tables

**Table 1:** Mechanical properties of aged PLA samples after 168 h storage as a function of storage temperature and RH.

		PLA			
Temperature [°C] Humidity [%]		23 °C	50 °C	70 °C	90 °C
maximum elongation (%)	10%	2.14 ± 0.04 d,e	2.22 ± 0.03 c,d	2.22 ± 0.03 c,d	
	50%	2.08 ± 0.03 e	2.38 ± 0.03 a,b	0.36 ± 0.13 f	
	75%	2.10 ± 0.02 e	2.48 ± 0.02 a		
	90%	2.09 ± 0.02 e	2.29 ± 0.09 b,c		
Young’s Modulus (MPa)	10%	3542.96 ± 83.0 c,d	3665.19 ± 60.3 b,c	3698.48 ± 65.7 b	
	50%	3596.30 ± 21.2 b,c,d	3512.54 ± 122.2 d	3934.72 ± 66.3 a	
	75%	3540.14 ± 10.0 c,d	3548.12 ± 30.6 c,d		
	90%	3546.43 ± 61.7 c,d	3578.21 ± 117.7 b,c,d		

The same letter means no significative difference. Batches without specified values could not be subjected to mechanical testing.

**Table 2:** Mechanical properties of aged PLA–starch samples after 168 h storage as a function of storage temperature and RH.

		PLA with 50 wt.% Starch			
Temperature [°C] Humidity [%]		23 °C	50 °C	70 °C	90 °C
maximum elongation (%)	10%	0.87 ± 0.10 c	1.07 ± 0.04 a	0.94 ± 0.01 b,c	0.49 ± 0.08 d
	50%	0.99 ± 0.02 a,b,c	1.01 ± 0.03 a,b	0.90 ± 0.06 b,c	
	75%	0.91 ± 0.07 b,c	0.89 ± 0.02 b,c	0.59 ± 0.11 d	
	90%	0.88 ± 0.02 c	0.90 ± 0.02 b,c	0.27 ± 0.07 e	
Young’s Modulus (MPa)	10%	4612.26 ± 58.66 a	4459.31 ± 53.72 a,b,c	4389.81 ± 43.99 b,c	4512.989 ± 212 a,b
	50%	4339.22 ± 19.06 c	4097.82 ± 83.53 d,e	3948.53 ± 48.33 e,f	
	75%	4140.70 ± 22.19 d	3770.14 ± 14.38 g,h	3616.86 ± 73.79 h	
	90%	3852.69 ± 38.38 f,g	3383.63 ± 71.42 i	2913.88 ± 147.6 j	

The same letter means no significative difference. Batches without specified values could not be subjected to mechanical testing.

## Data Availability

The data presented in this study are available on request from the corresponding author.
